# Understanding the genetic mechanisms and cognitive impairments in Down syndrome: towards a holistic approach

**DOI:** 10.1007/s00415-023-11890-0

**Published:** 2023-08-10

**Authors:** Yara Abukhaled, Kenana Hatab, Mohammad Awadhalla, Hamdan Hamdan

**Affiliations:** 1 Department of Physiology and Immunology, College of Medicine, and Health Sciences, Khalifa University, 127788 Abu Dhabi, United Arab Emirates; 2 Healthcare Engineering Innovation Center (HEIC), Khalifa University, 127788 Abu Dhabi, United Arab Emirates

**Keywords:** Down syndrome, Intellectual disability, Morphology, Neuron, Over-inhibition

## Abstract

The most common genetic cause of intellectual disability is Down syndrome (DS), trisomy 21. It commonly results from three copies of human chromosome 21 (HC21). There are no mutations or deletions involved in DS. Instead, the phenotype is caused by altered transcription of the genes on HC21. These transcriptional variations are responsible for a myriad of symptoms affecting every organ system. A very debilitating aspect of DS is intellectual disability (ID). Although tremendous advances have been made to try and understand the underlying mechanisms of ID, there is a lack of a unified, holistic view to defining the cause and managing the cognitive impairments. In this literature review, we discuss the mechanisms of neuronal over-inhibition, abnormal morphology, and other genetic factors in contributing to the development of ID in DS patients and to gain a holistic understanding of ID in DS patients. We also highlight potential therapeutic approaches to improve the quality of life of DS patients.

## Introduction

Down syndrome (DS) is the most common genetic cause of intellectual disability. It is caused by the presence of three copies of the Homo sapiens chromosome 21 (HSA21), rather than two. According to the CDC, DS can present in three forms: Trisomy 21, translocation DS, and mosaic DS. The classical form of DS is *Trisomy 21*, that about 95% of individuals with DS have. It is due to a mishap during cell division called “nondisjunction” [1]. This normally occurs before or at the point of conception; either the sperm or the egg would fail in separating their reciprocal HSA21. With this process, the embryo will have three copies of HSA21, rather than two. *Translocation DS*, which only 3% of people with DS have, occurs before or at the time of conception; a part of HSA21 becomes attached or ‘translocated’ onto a different chromosome [2]. Thus, children with this type of DS will have the normal two copies but would also gain the genetic material from the other HSA21 that is attached to a different chromosome [3]. The least abundant form of DS (2% of people with DS) is the *mosaic Down syndrome.* The term ‘mosaic’ implies a mixture of genetically different cells that developed from a single zygote [4]. Such individuals will have some cells with three copies of chromosome 21, and others with the normal count of two chromosomes. Moreover, individuals with the mosaic form of DS tend to present with relatively fewer features of DS.

### Clinical and phenotypical manifestations of DS

Our knowledge of the clinical and phenotypical manifestations of DS has drastically evolved since its discovery in 1959 [5]. This is greatly due to the various DS mouse models such as Ts65Dn that was first presented in 1990 [6]. Ts65Dn is a trisomic mouse for chromosome 16 and is greatly used to study DS given that it shares many of the trisomic features seen in humans. However, the Ts65Dn mouse model does have its limitations given that it is trisomic for 35 more genes than those presented on HSA21, and it lacks 75 trisomic genes that are normally seen in HSA21 [7]. Despite advances in creating more similar versions of T65Dn to human subjects, they end up being trisomic for a distinct subset of HSA21, but never the full HSA21 [7].

It is important to note that DS manifests with a wide range of phenotypes and can impair the normal functioning of a wide range of organ systems, such as the musculoskeletal system (MSK), the neurological system, and the cardiovascular system [8].

When considering the MSK system, pes planus is the most prevailing feature, in addition to delayed ambulation and arthritis [9]. They also commonly present with a short stature, muscle hypotonia, and atlantoaxial instability [10, 11]. As for *neurological system*, it is important to consider 3 different categories: neurodevelopmental, psychotic, and neurological [12]. Their neurodevelopmental status is stunted, and that is presented via their intellectual disabilities, developmental delay, language disorders, and cerebellar hypoplasia. Psychiatrically, these patients are more prone to anxiety and depression, along with behavioral disturbances. Whereas neurologically, DS patients are at an increased risk for Alzheimer disease and epilepsy [8]. Concerning the *cardiovascular system,* these patients are at an increased risk for congenital heart defects, especially atrioventricular septal defects (AVSDs) [13].

It is important to note that other manifestations may arise including hypothyroidism, autoimmune diseases, obstructive sleep apnea, hearing and vision issues, hematological disorders, and recurrent infections [8].

### Intellectual difficulties in DS patients

“Intellectual Developmental Disorder” as DSM-5 reinstates instead of “mental retardation” is a group of neurodevelopmental disorders that starts early on in childhood and is distinguished with intellectual difficulties, along with difficulties in conceptual, social, and practical areas of living. The diagnosis of ID, as claimed by the DSM-5, has to satisfy three criteria’s. These individuals would have deficits in intellectual functioning, adaptive functioning, and the onset of these occurs in childhood. Since DS affects intellectual functioning, they would have problems with abstract thinking, reasoning, planning, problem-solving, academic learning, and learning from experiences. They would also have difficulty with conforming to the developmental and sociocultural standards of society. In return, they would not be able to have personal independence and have the ability to handle social responsibility [14]. The DSM-5 also classifies ID severity into mild, moderate, and severe, and profound classified based on daily skills ranging from living independently with minimum levels of 24-h care and support.

When it comes to the ID endured by DS patients, it is classified as mild–moderate in severity given that that they have a wide range of disparities when considering their IQ, language, attention, memory, and functional abilities [8]. In addition, studies report that 1 in every 54 DS patients has been diagnosed with autism spectrum disorder (ASD) [15], as well as ADHD in 6% of the DS population [16]. Such comorbidities increase the severity of ID.

### Mechanism 1: morphological discrepancies of pyramidal neurons in DS mouse models and ID

Given that ID is the most prominent trait of DS, this paper aims to further indulge into the different mechanistic changes present in these patients that may correspond to their ID.

One mechanism of interest is the morphological changes of neurons in DS patients. The human brain contains billions of neurons that receive and send information encoded in electrical signals. Historically, research into human intelligence focused on the structure of the brain, and on genes associated with intelligence or with intellectual disability. However, little research has been devoted to understanding the association between the neuronal morphological discrepancies seen in DS brains and ID. Hence, studying the morphologic characteristics of diseased neurons on a cellular level can help achieve a better understanding of their pathology and give insights for further research into novel therapies.

In this paper, we focus on the morphology of pyramidal neurons (PN). This is because PNs are found in structures that carry out advanced cognitive functions and they are the most abundant cells in the mammalian neocortex. Hence, alterations in PNs morphology are bound to contribute to some forms of cognitive deficits. PNs are composed of a single axon that arises from the soma and branches into multiple excitatory synaptic contacts. PNs also have a dendritic tree, composed of basal and apical dendrites, that plays an important role in signal transmission. [17]. Although these are the generally defined characteristics of PNs, variations do exist. The characteristics of PN can vary widely based on their orientation and position in the cerebral cortex. Research has recently shown a correlation between intelligence quotient (IQ) scores and the dendritic structure of temporal cortical PNs [18]. Patients with DS have alteration and variation in their PNs [19], and hence, the integration and processing of information are clearly deficient in such patients. Through understanding these morphological variations, therapeutic approaches may be set in place to slow down or halt these alterations and therefore reduce the severity of ID.

### Mechanism 2: neuronal over-inhibition and ID

Another mechanism discussed in this review is neuronal over-inhibition. The activity of the brain is regulated by a balance between the excitatory and inhibitory activity of neuronal circuits. The loss of this normally maintained equilibrium can lead to a spectrum of disorders based on the severity of the dysregulation. The chief excitatory neurotransmitter in the nervous system is the amino acid glutamate which plays an important role in numerous physiological functions in neurons. The activity of glutamate is modulated by over 30 proteins that can be divided into membrane-bound receptors or transporters [20]. In an evolutionarily efficient process, glutamate can be converted by the enzyme l-glutamic acid decarboxylase to gamma-aminobutyric acid (GABA), the main inhibitory neurotransmitter of the central nervous system. It performs its actions by binding to ion-gated GABA-A receptors and metabotropic GABA-B receptors [21]. It is a major regulatory molecule of neuronal circuits, and offsets in its balance are involved in a multitude of neurodevelopmental disorders. The over-inhibition section of this paper will discuss the excitatory and inhibitory imbalances seen in the neurons of DS patients and explain the role these imbalances play in the pathophysiology of ID.

In the last 5 decades, the average life expectancy of DS patients has risen from an estimated 26 years in 1950 to 58 years in 2010, possibly due to the enhancement in childhood survival for patients with DS [22]. As the life expectancy of DS patients increases, it becomes necessary to find solutions for the debilitating ID that DS patients suffer from to improve their quality of life. Therefore, we believe that dissecting these two dominant realms of changes in a DS individual can then help in further discussing potential management tactics or pharmaceutical approaches, potentially ameliorating the neurodevelopmental symptoms.

This paper aims to discuss and review the mechanisms of over-inhibition and the morphological abnormalities seen in PNs within the brains of DS patients and to provide a more holistic approach to understanding the plausible causes of such cognitive impairments. We will also highlight the effects of trisomy 21 on genetic dosages and how such imbalances may even contribute to the over-inhibition theory and the abnormal neuronal morphologies. Finally, we will provide plausible therapeutic approaches to potentially reduce the severity of ID and improve the quality of life of DS patients. This field of research has witnessed rapid advancements in recent years. Hence, papers were selected based on the findings of the most up-to-date studies, studies with substantial findings, and papers that allow for a more comprehensive understanding and overview.

## Epidemiology

DS imposes a huge financial and social burden on family members and society, hence exploring the global trends and patterns of DS, as well as conducting regional stratifications is of great value to better comprehend and regulate DS. It is also worth noting that human aneuploidy is a very complex topic due to its multifactorial nature [23, 24]. Therefore, many aspects of DS such as environmental factors must be explored before understanding ways to manage it [23, 24].

### Prenatal screening and global demographic trends in DS

An estimated 30% reduction in the number of babies with DS in the US was identified between the years 2006 and 2010 due to elective pregnancy terminations. This has served a positive impact in alleviating the burden that comes along with having a child with DS [25]. This can be attributed to the increasingly widespread practice of prenatal screening. Since 1989, there has been an expansion and improvement in the availability of antenatal screening, and this has helped offset an increase in many of the birth defects we are currently aware of including DS [26]. Studies have shown that the recipients of antenatal screening were observed in 70% of mothers above the age of 37 and in 43% of younger mothers in Wales and England [26]. Studies also reported that antenatal screening occurred in 15% of mothers in the UAE and in Ireland [27]. This could partially explain why Ireland was in the top three countries with the highest prevalence of age standardized rates in both prevalence and incidence of DS [27, 28]. This can also be used to explain why the incidence of DS in the UAE is 2.2 per 1000 among UAE nations and 1.66 per 1000 among non-UAE nationals [29]. Research also determined a lower rate of prenatal screening from women of lower socioeconomic backgrounds [30]. This can be attributed to the lack of education and resources available to them. A Dutch study also found that Turkish and Moroccan women were less likely to participate in prenatal screening for DS [30].

Table 1 confirms a consistent association between the global demographic trends of DS from 1990 to 2019 and antenatal screening [31]. Ten studies (Table 1) showed a clear association between the descending birth rate of DS and prenatal screening. These studies once again amplify the importance of demographics on the availability of prenatal care and the presence of more sophisticated screening methods.

**Table 1:** Extracted from: Huete-García& Otaola-Barranquero, 2021 [31]

No.	Study	Region	Range	Percent change	Main cause
1	Lindsten et al. (1981) [153]	Sweden	1968–1977	− 18.5	Screening
2	Mulcahy (1983) [154]	Western Australia	1967–1981	− 20.2	Screening
3	Wilson et al. (1992) [155]	Los Angeles (USA)	1974–1988	− 36.8	Screening
4	Cheffins et al. (2000) [156]	South Australia	1982–1996	− 57.6	Screening
5	Lai et al. (2002) [157]	Glasgow (UK)	1980–1996	− 23.9	Screening
6	Siffel et al. (2004) [158]	Atlanta (USA)	1994–1999	− 17	Screening
7	Hei-Jen et al. (2005) [159]	Taiwan	1993–2001	− 65.2	Screening
8	Mendez-R. et al. (2014) [160]	Cuba	2002–2012	− 16.7	Screening/maternal age
9	Huete-García (2016) [161]	Spain	1976–2010	− 65.6	Screening
10	Jarurata-nasirikul (2017) [162]	Southern Thailand	2009–2013	− 38.9	Screening/maternal age

The white race seems to be more aware of the importance of prenatal screening and the potential ways of terminating pregnancies. Such distinctions among the different ethnic groups can be based on socioeconomic backgrounds, policies, provisions, religious beliefs, etc. [28]. The differences in incidence and prevalence of DS can be partially attributed to this variability as the termination of DS pregnancies undoubtedly decreases the burden [28].

### Risk factors of DS

It is well known that a major risk factor for DS is advanced maternal age (AMA), defined as a woman aged 35 years or older at the time of delivery [32]. The frequency of advanced maternal age has been increasing since the 1980s, as more women are having late childbirths [33]. In fact, a study showed that 10 out of 12 European countries reported more than 50% of their mothers are of advanced age [32]. This is important to consider, because despite advancing maternal age, widespread termination of pregnancy has established a relatively stable incidence of DS [28, 32, 34].

When considering AMA in other regions of the world, a study conducted in 2007 in the UAE suggested that the higher incidence of DS among UAE nationals (2.2 per 1000) compared to the incidence of DS in non-UAE nationals (1.66 per 1000) is mainly due to AMA, with more continuing to bear children until their 50 s [29]. Such findings are different than in regions like Europe due to religious beliefs on matters such as pregnancy termination. Hence, in such cases, introducing better and mandatory prenatal screening can drastically reduce such incidence rates.

Another important regional difference to be examined is consanguineous marriages, a common practice in multiple areas of the world, such as North Africa and the Middle East [35]. Consanguineous marriages are a major cause of higher incidence of DS in certain regions due to the increased expression of autosomal recessive genetic mutations [27, 36]. It has also been reposted that consanguinity amplifies the frequency of cardiac malformations within DS patients [36]. Studies also took advantage of large and unique populations to compare the profile of meiotic errors and recombination patterns in consanguineous and non-consanguineous marriages. Among such meiotic errors, the overwhelming majority were of maternal origin, and they were found to be significantly more common in consanguineous marriages than non-consanguineous marriages (74% and 10%, respectively). Again, this highlights some of the genetic burdens that come with consanguineous marriages and the significance of the maternal genes in contributing to such abnormalities [36].

In general, understanding regional differences is crucial to regulate and better help families with DS members. Despite relatively consistent patterns of incidence in DS worldwide, the incidence in some countries continues to be of concern. This can be due to the lack of education, resources, and prenatal screenings. Through such studies, we can also provide better genetic counseling and introduce better prenatal diagnostic services and antenatal screening programs and, hence, alleviating the psychological and social burdens imposed on family with DS patients.

## Morphology

Neurons, the primary component of the CNS, are electrically excitable cells that can receive, transmit, and integrate information via electrical and chemical signals within circuits. A big challenge in neuroscience revolves around trying to gain a comprehensive understanding of the morphological heterogeneity of neurons and how the morphology of the neurons plays a role in dictating its function. Not surprisingly, the pioneering work of Ramon Y Cajal since the emergence of neuroscience which focused on classifying and categorizing neurons through Golgi staining is still proceeding. This helped us better understand neuronal morphology, which served as a key determinant of informational processing in the nervous system. The repertoire of morphological variations allowed for differences in signal transmissions, circuits, integrations, and connections.

To understand the brain, studying morphology and organization is crucial. Two long processes that primarily characterize the neuronal structure are the axons and the dendrites. They are essentially conduits needed to generate and integrate electrical and chemical signals. Their morphology will dictate how these impulses are to be integrated and transmitted. Before focusing on the specificities of neurons and the differences observed between euploidy and Down syndrome patients, it is important to understand that regional brain differences in Down syndrome patients have been extensively observed and studied. Through various imaging techniques such as MRI, subjects with Down syndrome were found to have overall smaller brain volumes, and the greatest discrepancy was characterized in the frontal lobes, brain stem, and the cerebellum [37, 38]. Findings also pointed to Down syndrome brains having a disproportionately greater subcortical gray matter volume and a smaller cerebellar volume. Such abnormalities from an early age can help explain the observed cognitive and developmental deficits we see in Down syndrome patients [39].

Moreover, electroencephalogram (EEG) studies identified that the brains of Down syndrome patients lacked normal alpha amplitudes in central, parietal, temporal, and occipital sources [40]. This suggests impaired cortical neuronal synchronization and hence impaired neuronal functioning. An fMRI study also identified the absence of normal receptiveness in the language areas of Down syndrome brains during passive story listening [41]. Hence, the integration and processing of information is clearly deficient in Down syndrome patients. A successive study also determined atypical neural activation (both qualitatively and quantitatively) than typically developing patients of matched chronological ages [42]. Additionally, it is important to remember that variation can also exist between age cohorts with Down syndrome. Analysis performed on DS infants less than 6 months of age indicated a higher quantity of connections in layer 3 compared to the same cohort after 6 months of age as the opposite was found, and such results indicate that there is neuronal growth cessation and dendritic atrophy very early on in life [43].

More recent studies have focused on understanding neural correspondence to intellectual disabilities in Down syndrome patients. The neuron we chose to focus on is the pyramidal neuron. This is because pyramidal neurons are the most frequently observed neurons in the neocortex implying their importance in the processing of information. In recent years, pyramidal neurons were shown to possess a great deal of physiological and behavioral differences across different cortical layers despite the apparent morphological heterogeneity [44, 45]. Pyramidal neurons were also seen to express the increasing size of spines and dendritic trees as they progress to higher order areas [46]. Such regional differences and connectivity help establish a hierarchy in information processing. Hence, it is definite to say that alterations in pyramidal neuron structures can alter information processing and consequently contribute to intellectual disabilities.

Before indulging into the types and alterations of pyramidal neurons, it is important to remember that dendritic spines are dynamic structures. They constantly undergo changes in shape, size, and density [47]. Part of dendritic growth is regulated by genetic factors which is why it is important to understand its association with disorders like Down syndrome. The other important regulator of dendritic growth is neuronal activity as research has shown a strong association between environmental enrichment and arborization [48].

### Pyramidal neurons

Thick-tufted pyramidal neurons are one of the most extensively studied pyramidal neurons in the neocortex and have become the standard for understanding information processing [49]. These thick tufted pyramidal neurons can be found in the deep portions of layer V [45, 49]. Thick tufted pyramidal neurons are primarily characterized by a thick tufted apical dendrite with oblique dendrites emerging from the main apical dendrites before bifurcating into the tuft dendrites. It is important to note that layer V pyramidal neurons possess many basal and apical dendrites with more frequent distribution on the basal end. These basal dendrites receive input from nearby neurons and layer II/III pyramidal neurons [50, 51]. Even within layer V pyramidal neurons, variability exists as some pyramidal neurons were classified as having slender apical dendrites in the superficial region of the layer [52]. Layer VI pyramidal neurons were seen to project into the thalamus and other cortical areas and are the only pyramidal neurons that do not bifurcate in layer I [53, 54]. Another important set of pyramidal neurons are those of layer II/III, which provide cortico-cortical connections and hence function to integrate information across cortical areas and hemispheres [55].

Dendrites of pyramidal neurons are covered with spines that receive synaptic input, and the density of synaptic inputs significantly differs in different cortical areas. They also appear to be regulated differently in response to hormones and those with neurological illnesses [56, 57]. From the discussion above, neuronal morphology plays a drastic role in neuronal processing and computation. Therefore, it is essential to study such pathological variations in neuronal morphology to better treat neurodevelopmental disorders and understand the importance of the structure–function relationship.

**Fig. 1:**
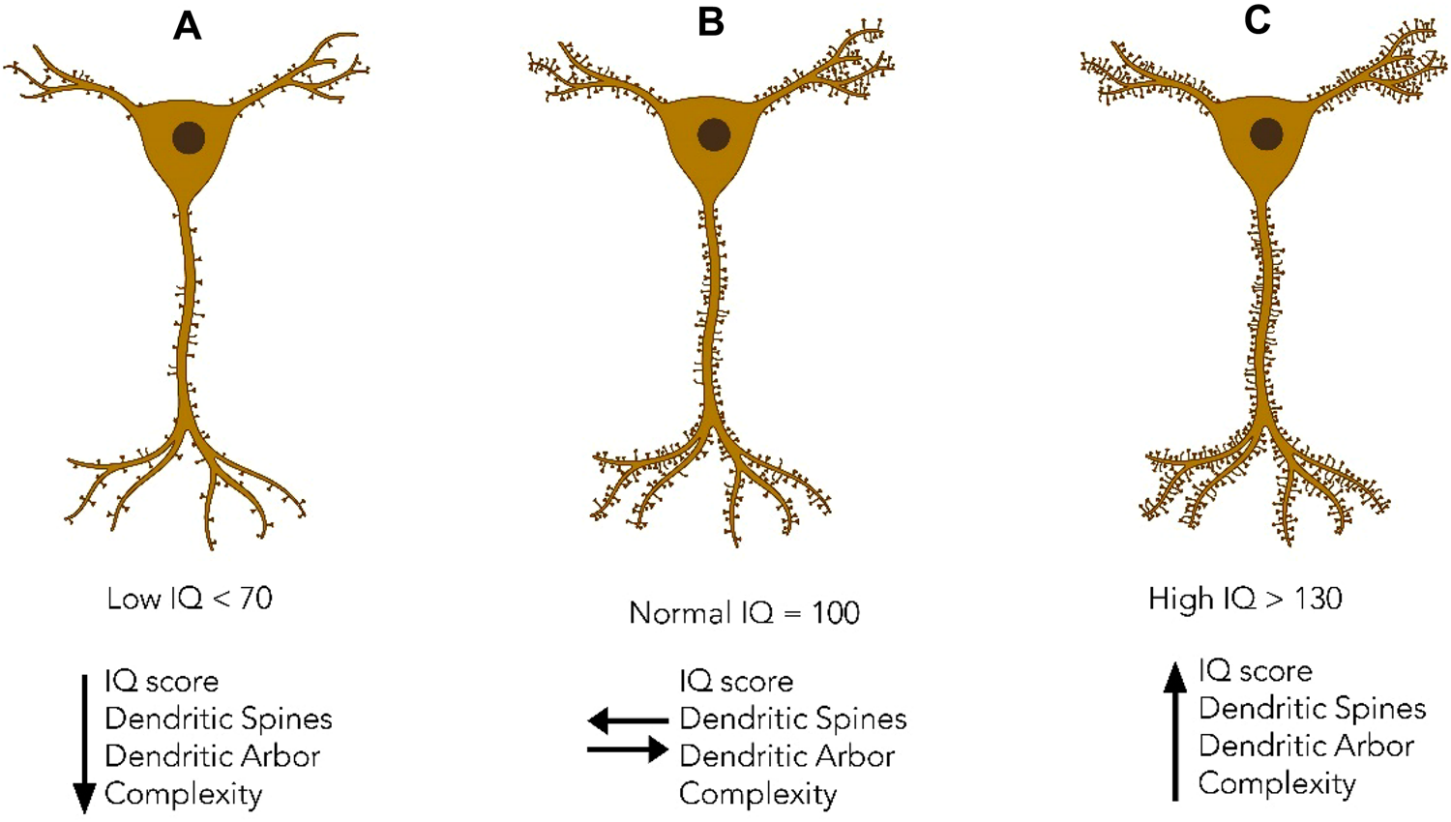
The following figure was created to illustrate how both dendritic spines and arbors contribute to shaping the complexity of neuronal networks. Through various studies, a positive correlation was established between complexity and cognitive abilities

Analysis of pyramidal neurons in the motor cortex of patients with Down syndrome showed a reduction in the number of spines and spines being abnormally too long or too short. Such findings are believed to be associated with motor incoordination and mental retardation [58]. Figure 1 illustrates how the complexity of dendritic spines and arbors are positively correlated to cognitive abilities such as attention, working memory, and spatial learning. Studies have also expanded on these findings and identified a reduction in the number of dendritic spines in pyramidal neurons of the hippocampus of young patients with Down syndrome [59]. Additionally, basilar dendrites of cortical pyramidal neurons appeared to be shorter than usual in subjects older than 4 months old [60]. However, quantitative analysis of layer IIIC pyramidal neurons of the prefrontal cortex in 2.5-month-old infants revealed no alterations in dendritic differentiation between euploid and DS cases [61]. Such findings suggest that DS patients begin their lives with normal pyramidal neuron morphology in layer III and it is only after 2.5 months of age do these pathological changes occur. Successive to this age, there is a steady decrease in such parameters, especially in the apical dendrites [62]. Moreover, a significant reduction in the number of dendritic spines was found in the basilar and apical dendritic arbors of CA1, CA2, and CA3 regions of pyramidal neurons in the hippocampus when compared to aged, matched controls [63]. Layer III and V pyramidal neurons of the parietal cortex were also seen to possess more degenerative changes when compared to the standard pyramidal neuron of the same regions [64]. When combined, we can see that alterations in the dendritic spines of pyramidal neurons are evident in DS patients.

In the present studies, the basal dendrites of layer III pyramidal neurons within the frontal cortex were found to be smaller, less spinous, and less branched than the controls [65]. Moreover, environmental enrichment had less effect on the structure of pyramid neurons in Ts65Dn mice [65]. Hence, early implementation of special care programs may not be as effective as initially believed. Additionally, using Lucifer yellow to visualize the basal dendritic tree of pyramidal neurons, they were identified to be smaller, with fewer branches, and 24% less spinous in T265Dn mice than in the control groups, which was mirrored in human subjects [66]. More recent evidence also pointed to dendritic branching defects in the neocortical pyramidal neurons of 2-day-old Ts65Dn pups [67]. Age-affected dendritic alterations were also noted as dendritic branching defects involving the basal domain occurred shortly after birth, whereas the apical domain alterations occurred shortly after [67].

### Gene expression and neuronal morphology

In the past decade, there has been an increasing interest in trying to understand the association between the extra HSA21 copy and the presentation of DS patients [68]. Among the genes of interest, DYRK1A has received increasing attention. DYRK1A encodes for dual-specificity tyrosine-(Y)-phosphorylation-regulated kinase 1A protein and has been associated with neurodevelopment [69]. Research has identified that DYRK1A is overexpressed to about 1.5-fold at the adult and fetal stages of life [70]. This overexpression was noted throughout the cortex. It is worth noting that insufficiency of DYRK1A can also contribute to a myriad of neurodevelopmental defects, such as intellectual disabilities, mental retardation, and microcephaly [71–73]. The overexpression of DYRK1A also appeared to affect the morphology of pyramidal neurons differently based on the cortical area being studied. For example, overexpression of DYRK1A largely increased the number of spines on oblique dendrites of pyramidal neurons in deep layers [74]. Other studies pointed to DYRK1A overexpression and under-expression both reduced basal dendritic spine density [75, 76]. Regardless, all available data show the need for adequate DYRK1A to establish and maintain appropriate dendritic arborization. Additionally, all data converged to a tight relationship between DYRK1A expression and spinal abnormalities [74–76].

From gestation to adulthood, treating DYRK1A transgenic mice with epigallocatechin gallate (EGCG), a DYRK1A inhibitor, has been shown to improve long-term memory [77]. Furthermore, a 4-week treatment program with EGCG in 3-month-old and 4-month-old DYRK1A transgenic mice improved the spine density of prefrontal cortex pyramidal neurons and normalized the long-term potentiation (LTP) [78]. This shows a strong relationship between the degree of expression of DYRK1A and the effectiveness of pyramidal neuron function. Additionally, a recent clinical trial with EGCG helped improve visual recognition memory, working memory performance, and adaptive behavior in young patients with DS by giving them EGCG [79]. Through more research and more clinical trials, we can determine better and more effective treatments that can help improve the brain functioning of Down syndrome patients.

*RCAN1* (previously called *DSC1*—Down syndrome critical region gene 1) is another gene located on HSA21 and is found to be upregulated in DS patients by up to 1.9-fold in the fetal brain and up to threefold in the adult brain [80, 81] RCAN1 was found to be overexpressed in regions of neural proliferation and differentiation zone [80, 81]. Functionally, *RCAN1* is believed to be a modulator of calcineurin by encoding for a negative regulator VEGF-calcineurin. Calcineurin is needed for various cellular functions, such as T-cell activation and proliferation and the formation of new memory [82, 83]. RCAN1 overexpression in DS mice models was found to not affect on the overall brain volume but rather had an effect on a neuronal level. RCAN1-TG apical dendrites of CA1 pyramidal neurons exhibited a 39.2% reduction in apical spine density and a 28.8% reduction in the basal spine density [83]. This shows that over-expression of RCAN1 has a huge effect on the overall morphology of pyramidal neurons [83].

## Over-inhibition

Excitatory and inhibitory neurotransmitters heavily regulate neuronal cortical and subcortical circuits, and the disruption of the balance between excitation and inhibition is a proposed etiological mechanism for a wide range of neurodevelopmental disorders [84, 85]. At a neuronal level, the balance between excitatory and inhibitory neurons is important for processing and transmitting information; therefore, such processes must remain well regulated and controlled. Based on to date research, there are several key regulators to maintain this balance, such as intrinsic neuronal excitability, neuronal plasticity, and synaptic transmission [86–88]. On a more cellular level, glutamatergic excitatory neurons and GABAergic inhibitory neurons are the key contributors to balance [86–88]. GABA is the primary inhibitory neurotransmitter in the developed brain. Given the inhibitory effect of GABA on the brain, an excess of GABA in a mature brain can result in sedation, while GABA synapse blockade can result in seizures. GABA performs its actions by binding ion-gated GABA-A receptors and metabotropic GABA-B receptors [21]. GABA-A receptors are ligand-gated chloride channels that are composed of five subunits. A total of 19 possible subunits have been identified, with the most common isoform consisting of two alpha subunits, two beta subunits, and a gamma subunit [89]. When GABA is released into synaptic clefts and binds to GABA-A receptors, it generates a current dependent on the difference between the equilibrium potential of chloride anions (Cl^−^) and the resting potential of the postsynaptic neuron (Vm). The intracellular Cl concentration is an important factor in determining the direction and magnitude of current through the GABA-A receptor [90]. GABA/GABA-A receptor signaling is the most significant inhibitory pathway in the central nervous system [89]. GABA can also bind to GABA-B receptors. The GABA-B receptor is composed of two subunits: GABA-B1, which is responsible for ligand binding, and GABA-B2, which mediates G protein interactions [91]. Although less is known about the function of the GABA-B receptors, studies have already shown that they are involved in postnatal inhibitory circuit organization. GABA-B receptors are also involved in neurogenesis in developing and adult brains [92]. Given that a range of amino acids and monoamines are necessary for proper brain development, assessing the alterations and differences in Down syndrome brains is necessary. This will not only help address the plausible underlying causes of ID in DS patients but also help determine better pharmacological approaches.

Neuronal dysfunction in DS begins as early as the embryonic stage, as fetal DS brains exhibit abnormal levels of neurotransmitters critical for normal brain development [93]. In developing neurons, GABA acts as an excitatory neurotransmitter. This is because the intracellular Cl^−^ concentration of immature neurons is higher due to early expression of Na^+^-K^+^-2Cl^−^ co-transporters (NKCCs), which import Cl^−^ intracellularly, and delayed expression of K^+^–Cl^−^ co-transporters (KCCs), which export Cl^−^ extracellularly [94]. In rodent brain studies, a transition in the function of GABA from an excitatory to an inhibitory neurotransmitter happens by the end of the second postnatal week due to the increased expression of the neuronal Potassium–Chloride channel KCC2 [94, 95]. The control of intracellular Cl^−^ homeostasis by KCC2 results in relatively reduced intracellular Cl^−^ in mature neurons, allowing GABA to perform hyperpolarization and inhibitory signaling [96]. A study performed in the Hospital Duran I Reynals on fetal frontal cortex brain tissue of male fetuses with DS showed that the concentration of GABA was reduced by 61% in subjects with DS [93]. This deficiency of GABA in the developing brain can lead to over-inhibition by loss of excitatory synaptic transmission. Additionally, GABA is an important factor that controls dendritic maturation, so a deficiency in GABA can impair the maturation of the developing brain [97].

In addition to neurotransmitter deficits during fetal development, the brains of DS patients are also affected by alterations in the neuronal balance. Studies performed by proton magnetic resonance spectroscopy found that GABA/Cr ratios are decreased in children with DS, indicating that GABAergic over-inhibition in DS is not due to an excess of the neurotransmitter  [98]. A decrease in the GABA levels was attributed to the reduction in the number of GABA neurons within the temporal lobes of DS children. Such a reduction was attributed to the destructive process that occurs in the brains of DS patients [98, 99]. According to many studies, apoptosis is more intensified in the brains of DS patients, and many even confirmed a greater level of free radicals in the brains of DS patients [100, 101].

### Varying GABA levels in DS brains

Long-lasting changes in synaptic strength were a proposed mechanism to be the building blocks of learning and memory. Hence, many studies focused heavily on determining synaptic plasticity variations between Ts65n mice and the euploidy controls. Experimental approaches determined that hippocampal slices from Ts65Dn models revealed a deficiency in LTP in CA1 synapses [102, 103]. Additionally, through low-frequency stimulation of Schaffer collaterals (CA3 pyramidal neurons projecting to area CA1) have a more enhanced long-term depression effect (LTD) than when compared to their euploidy controls [103, 104]. Studies have also extrapolated these results to other brain regions, such as the dentate gyrus, and attributed these findings to excessive GABAergic activity [103, 105].

Different lines of evidence actually determined a connection between LTP, GABAergic overexpression, and learning deficits. One of the first pieces of evidence was found by incubating Ts65Dn hippocampal slices with Picrotoxin, a GABAA channel blocker which restored LTP [106–109]. Additionally, LTP was restored in the dentate gyrus via the chronic administration of a non-competitive GABA A antagonist Pentylenetrazole at non-epileptic doses [110]. These findings confirm the involvement of GABA A circuits in establishing LTP in DS mice. Furthermore, RO4938581, a negative allosteric modulator of a5-containing GABA A receptors, was also shown to improve LTP in the hippocampus of DS patients [111]. Interestingly, a study conducted by Roncacé determined no LTP alterations when using Picrotoxin within the perirhinal cortex. However, using CGP55845 (a GABA B antagonist) increased LTP magnitude much more significantly than those measured in euploidy controls. Such findings can be attributed to the varying locations of the brain being studied. It also amplifies just how difficult understanding over-inhibition is. However, these findings confirmed that LTP can be improved by inhibiting excess GABAergic activity.

### Increased GABA concentration in the synapses

Over-inhibition was also believed to be attributed to the elevated concentrations of GABA at the synapses in the brains of DS patients. Such concentrations can be detected either via biochemical methods or imaging techniques. As mentioned earlier, GABA levels in the temporal lobes of children with DS were determined to be lower via magnetic resonance spectroscopy (MRS) [99]. A recent study detected no differences in GABA levels between Ts65Dn and euploid mice within the hippocampus. When using biochemical methods, GABA levels were reduced by 60% in the frontal cortex of DS fetuses [94]. However, no differences in GABA concentrations were detected in the frontal cortex from postpartum DS patients [112]. When looking at other brain locations, such as the hippocampus, GABA was significantly reduced in DS patients [113]. Such findings were attributed to the loss of GABAergic neurons in the brain's cortical regions. Additionally, given all these findings, it is unlikely that over-inhibition is due to a greater GABA concentration.

### GABAergic neuron levels in DS patients

Over-inhibition can also be attributed to the number of GABAergic neurons and the alterations in synaptic connections. During development, glutamatergic and GABAergic neurons are generated in separate locations. Approximately one in five neurons in the neocortex of adults are inhibitory and uses GABA as a neurotransmitter to hyperpolarize the target postsynaptic neuron [114]. This proportion is believed to be preserved through adulthood [114]. Interestingly, research found that neuronal progenitor cells (NPCs) from the periventricular zone of new-born Ts65Dn mice overexpress dual-specificity tyrosine-(Y)-phosphorylation-regulated kinase 1A (Dyrk1A) by around 1.5 folds [115]. The product of the Dyrk1a gene is the tyrosine-regulated kinase DYRK1A. DYRK1A is involved in brain development. It controls neural cell differentiation and synaptic function [116]. DYRK1A also plays specific roles in the adult central nervous system [117]. Dyrk1a is located on HSA21, so an extra copy of the chromosome means an enhanced expression.

Dyrk1A is also believed to promote premature neuronal maturation of the trisomic NPCs and enhance GABAergic differentiation compared to the disomic NPCs [115]. Studies also found that the neuronal density in the CA1 region of the hippocampus is drastically reduced, neurogenesis was impaired, and synaptic connectivity was reduced in CA3 and CA1 regions of Ts65Dn mice [118–120]. Despite these changes, GABAergic connections were found to be reorganized in Ts65Dn mice at sites with reduced dendritic connections to augment these connections [105, 108]. GABAergic interneurons were also found to be at an increased number in the hippocampus and cortex of Ts65Dn mice [121, 122]. These interneurons play a vital role in modulating and fine-tuning neuronal activities. Hence, overexpression may influence the normal function of inhibitory circuits and contribute to the cognitive deficits observed in DS patients. Furthermore, research found that recovering euploidy phenotype can be achieved by restoring gene dosing of olig-1 and olig-2 from 3 to 2 copies [121]. Olig-1 and Olig-2 are key transcription factors needed and involved in stem cell differentiation [121, 122]. This helps establish a connection between genotypic and phenotypic alterations.

### GIRK2

Another gene located on HSA21 and indicated in DS neuronal alterations is the potassium inwardly rectifying channel subfamily J member 6 (KCNJ6). KCNJ6 encodes the G protein-activated inward rectifier potassium channel 2 (GIRK2). GIRK2 channels are selective for potassium ions (K^+^). The opening of these channels causes hyperpolarization and reduced neuronal excitability. They can thus alter the excitation–inhibition balance by impairing dendritic excitability [123]. A study performed on Ts65Dn mouse brains found that gene dosage overexpression of GIRK2 measured 1.5-fold higher levels of GIRK2 mRNA in Ts65Dn mouse brains compared to diploid mice [124].

### LTP and potassium/chloride channels

DS patients generally have a higher risk of seizures. This is counterintuitive if GABAergic over-inhibition is believed to be an underlying cause of many of their defective neuronal activity. Studies explained these contraindications by investigating the pertinent chloride concentration dysregulation throughout the cell upon GABA-A-R activation. Gramicidin-perforated whole-cell recordings were used to study the directions and ECl of GABA-A-R Cl^−^ currents. They concluded that WT mice had a higher intracellular Cl concentration (− 62.4 mV) in relevance to its resting membrane potential (− 66 mV) with the introduction of GABA, indicating the predictive inward current of Cl^−^ with the activation of the receptor. However, the opposite was present in Ts65Dn mice (resting potential = − 64.4 mV and ECl = − 58 mV), indicating Cl’s predictive outward current with the receptor's activation [125].

ECl is mainly set on the intracellular Cl concentration, which is dependent on the antagonistic actions of the Cl importer, NKCC1, and the Cl exporter, KCC2. Via western blotting, a recent study indicated the overexpression of NKCC1 in the entirety of the hippocampus and the CA3–CA1 subregion of the Ts65Dn mice in comparison to the WT mice, with no direct changes in the chloride exporters, KCC2 (*p* = 0.03) [125]. In addition, due to the dependence of NKCC1 and KCC2’s function on their location at the cell membrane, subcellular-fractionation experiments were done to detect the heavy overexpression of NKCC1 in the synaptosomal membrane fraction of the tissue belonging to the Ts65Dn mice and human subjects with DS (*p* = 0.043 and 0.023, respectively [125]. This indicates that chloride ions are accumulating intracellularly, shifting the reversal potential (ECl) towards a more depolarized state and dampening the efficacy of GABA-mediated inhibition in adult Ts65Dn mice [126].

The reversal effect was achieved using an NKCC1 blocker, bumetanide, which helped improve cognitive performance. This indicated that the shift of ECl was indeed accountable for the excitatory GABA-A-R signaling in the adult Ts65Dn mice. Furthermore, since excess GABA-A-R signaling was linked with compromised synaptic plasticity in Ts65Dn mice, adding bumetanide could completely rescue their LTP, returning it to the level observed in WT mice (*p* = 0.05) [125]. These findings provide an interpretive framework for past and currently ongoing research, demonstrating that GABA could be excitatory in adult Ts65Dn mice. It also provides a new therapeutic approach to help cognitive impairments in DS patients [125]. However, more detailed exploration is needed to understand GABA and chloride concentration in various brain regions and neurons to better understand cognitive impairment in DS brains.

### Dscam

Down syndrome cell adhesion molecule (Dscam) is a member of the immunoglobulin superfamily of cell adhesion molecules (Ig-CAMs) and is highly associated with the central and peripheral development of the nervous system [127]. It is an evolutionarily conserved type 1 transmembrane protein with functions, such as cardiac and neural development [128]. Given their important role, Dscam expression levels are generally tightly regulated. Research pointed to the fact that dysregulation of Dscam is a critical contributor to the pathogenesis of neuronal over-inhibition in DS mice. A study conducted by Liu et al. (2020) focused on the effect of Dscam on Chandelier cells (ChCs), given that ChCs are the most potent inhibitory neurons within the neocortex and play a critical role in the regulation of pyramidal cells [128]. Compared to heterozygous mice, Dscam-mice had the total cartridge length of each ChC to be reduced by 23%. Moreover, the size and number of the presynaptic boutons decreased by 16% and 20%, respectively [128]. Such information proved the importance of Dscam in ChC development. The loss of Dscam impaired GABAergic inhibition of pyramidal neurons. Next, they determined the effects of axonal cartridges in Ts65Dn mice, where Dscam is present in three copies. Compared to euploidy mice, the number and length of axonal cartridges were significantly greater in T655Dn mice by 28% and 11%, respectively [128]. Additionally, the average size of presynaptic boutons was enlarged by 21%, and the bouton number for each ChC. Through the normalization of Dscam levels (ex: Dscam + / ±), ChC presynaptic overgrowth was hindered, proving that overgrowth is mainly attributed to overexpression of Dscam.

### Variations in glutamatergic activity/synapses in DS patients

**Fig. 2:**
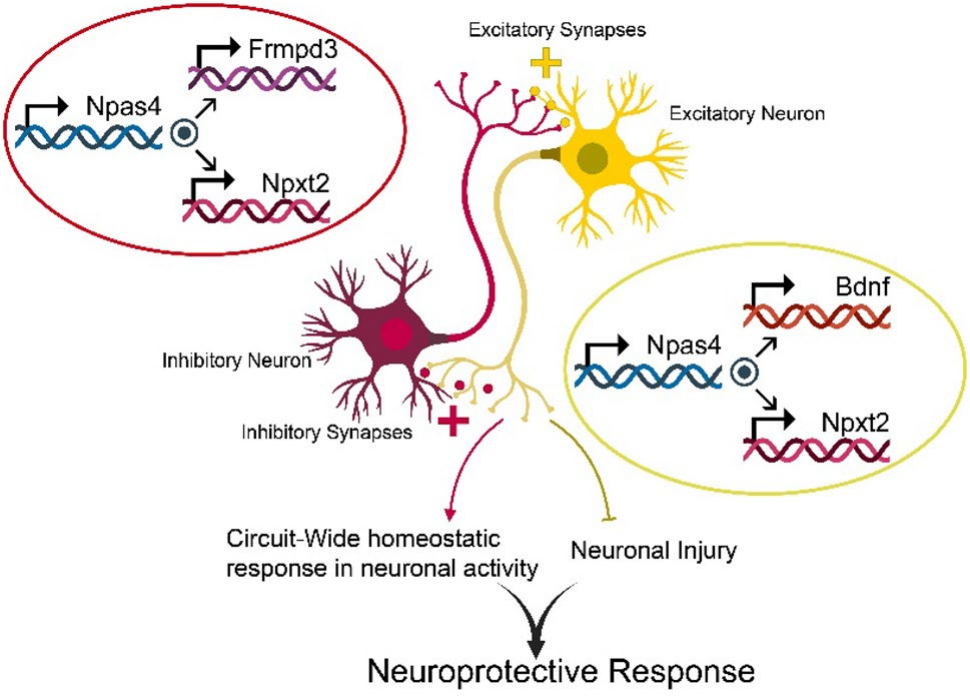
The following figure was designed to illustrate the importance of having the correct genetic dosage. The Npas4 gene was determined to play a crucial role in the cognitive deficits observed in the DS patients. Npas4 is expressed in response to various stimuli and is important in synaptic activity, plasticity, and memory formation. The overexpression of Npas4 in the brains of DS patients was shown to decrease long term potentiation and impair the ability of neurons to form and maintain connections. Retrieved from: 10.3389/fnins.2020.603373

Glutamatergic activity and transmission were also extensively studied to determine a potential relationship with behavioral and cognitive deficits in DS patients. MRS detected a significantly lower glutamate concentration in Ts65Dn mice compared to normal disomic controls [129]. The glutamate deficiency was accompanied by an NMDA receptor one mRNA and protein expression reduction [129]. Together, these alterations caused enhanced synaptic inhibition through paired-pulse analysis [129]. This is attributed to the offset in balance between glutamatergic and GABAergic activity. Thus, these findings suggest that the deficiency in glutamatergic expression plays a significant role in causing ID in DS patients. Through studying Down syndrome mouse models, research also identified a deficiency in SNX27 [129]. SNX27 is a protein-coding gene that helps maintain glutamate receptors on the surface of neurons. Hence, a deficiency in SNX27 means that the proper activity of neurons is hindered, and glutamate receptors cannot be maintained [129]. They were also able to deduce that C/EBPβ was lacking. C/EBPβ forms a family of transcription proteins needed to properly express SNX27 [130]. C/EBPβ is believed to be lacking, because it is negatively regulated by a microRNA called MiR-155. MiR-155 is encoded by chromosome 21, and since DS patients have an extra copy of chromosome 21, MiR-155 is upregulated, thereby reducing the expression of SNX27 [130]. This again emphasizes that genetics is highly associated with phenotypic expression; hence, more research is needed to grasp the association for a more holistic view (Fig. 2).

## Genetics

Due to the triplication of chromosome 21, a lot of genes show dosage effects increasing their expression in the cells or tissues of DS patients. The dosage effect is when the phenotype is a direct result of the overexpression of the structural gene [131]. In a study done in 2007 by Yahia-Graison and his colleagues, 29% of genes were overexpressed in DS cells, 22 followed the gene-dosage effect, while 7% were beyond that measure. They also witnessed an increase or decrease in the transcription of some genes to compensate for their overexpression [132].

Even though there are multiple genes overexpressed in trisomy 21, not all will cause biological harm to the body due to its lethal consequences [133].

*Dyrk1A*, a dual specificity tyrosine phosphorylation-regulated kinase 1A, is a protein-coding gene present on chromosome 21q22.2 [134]. In adult mice transgenic for Dyrk1A, Thomazeau et al. showed that its overexpression leads to an increase in the number of spines on oblique dendrites of pyramidal neurons in the prefrontal brain.

*OLIG1/2*, oligodendrocyte transcription factors, are co-expressed in neural progenitors. Both overexpression leads to defective neurogenesis in Ts65Dn mice [135].

*EURL* is a protein-coding gene present on 21q.21.1. Li et al. (2016) reported modification of the EURL mRNA levels impaired the progenitor proliferation, neuronal differentiation, and dendritic spine densities of cortical neurons.

*ERG* is a nuclear transcription factor present in 21q22 [136]. In mouse models, the triplication of ERG caused the reduction of prenatal cortical neurogenesis [137].

*SNX27*, an actin-sorting nexin 27, is an endosomal sorting machine that recycles and preserves cell surface receptors [138]. SNX27 also interacts with ionotropic glutamate receptors and offers protection to neurons from excessive glutamate [130, 139]. Wang et al. conducted a study that shows that SNX27 expression is reduced in DS patients due to the overproduction of *miRNA-155*, a post-transcriptional regulator of gene expression. It is encoded by the BIC gene located on chromosome 21 [140]. In their study, it was shown that miR-155 downregulates the transcription factor for SNX27, C/EBPβ, leading to a decreased synaptic recycling of glutamate, along with learning and memory decay.

## Therapeutic approaches

### Chromosome correction

Multiple experimental approaches have been designed to help correct the intellectual abnormalities occurring in DS patients by targeting the extra chromosome itself. Several techniques were successful in eliminating an extra Hsa21 in trisomic cells. They were all formulated to generate induced pluripotent stem cells (iPSCs). iPSCs are cells obtained from adult somatic cells that can be genetically wired to an embryonic stem cell-like state. This occurs through the forced expression of genetic factors that are vital for preserving the defining features of embryonic stem cells [141]. These studies managed to generate iPSCs from the fibroblasts of adults with DS.

Li et al. [142] generated iPSCs and added a Thymidine Kinase and Neomycin Resistance (TKNEO) fusion transgene at the locus of 21q21.3 of APP in one copy of Hsa21 via a modified adenovirus. The APP gene was chosen due to its location on the long arm of chromosome 21 and its increased expressivity of iPSCs. Twenty-two out of thirty-three of clones were found to have a spontaneous loss of an entire Hsa21 with no reported damage to other chromosomes. Other clones had point mutations, epigenetic silencing, and TKNEO deletions. Interestingly, disomic cells were found to proliferate faster than trisomic cells in the co-culture. Disomic cells were able to double their size on an average of 37 ± 0.7 h versus their trisomic counterpart, 47 ± 0.09 h.

Another study by Jiang et al. [143] thought of inserting X-inactive Specific Transcript (XIST) transgene into iPSCs obtained from males with DS. XIST is produced exclusively from the inactive X chromosome in women. XIST provides dosage equivalence between males and females by transcriptionally silencing one of the pairs of the X-chromosome [144]. However, they inserted the transgene at locus 21q22 of the gene DYRK1A in one copy of Hsa21. Of the clones treated, 85% of the chromosome was silenced with no alteration of other chromosomes. Similar to what occurs to the silenced X-chromosome, a chromosome 21 Barr body was noted. A few sub-colonies showed cells where one, two, or even three Hsa21 fusing with XIST RNA. Further testing showed that XIST may induce a robust dosage compensation of some of the genes overexpressed in Hsa21. Like the experiment of Li et al., disomic cells proliferated at a higher capacity than trisomic cells. However, it is unknown if the natural phenomenon of X-inactivation would occur normally if this experiment were to be applied using fibroblasts from females with DS.

An additional study by Amano et al. [145] used ZSCAN4 (zinc finger and scan domain) to normalize the karyotype of mice genetically engineered to become aneuploid or polyploid. ZSCAN4 is an embryonic stem cell-specific transcription factor that is needed to regulate pluripotency in embryonic cells. It binds to telomeres and regulates their elongation, aiding in embryonic cells' genomic stability [146]. Amano et al. encoded ZSCAN4 using a Sendai virus vector. They tested ZSCAN4 on iPSCs from fibroblasts of DS individuals. Up to 24% and then 40% of cells had normal karyotypes after only a few weeks. They assumed that ZSCAN4 can detect the unpaired chromosomes during cell division and detach them from the rest.

A novel study was done by Inoue et al. [147] derived amniotic fluid from a female fetus with DS at week 29 of gestation and established independent iPSC lines, where all cell lines in the iPSCs contained Hsa21 trisomy. Hsa21 diploids were observed with continuous culturing of iPSCs for 70 weeks. Based on gene chip analysis performed on the diploid and trisomy 21 iPSCs, the diploid iPSCs showed decreased expression levels of DYRK1A, SOD1, ETS2, APP, and DSCR1 by two-thirds. The author speculates the spontaneous reversion to disomy may be due to mitotic chromosome nondisjunction during the 70-week iPSCs cultivation.

The results obtained from these research studies suggest unattainable clinical applications. However, they do offer a new approach to understanding the mechanistic pathways of DS.

### Pharmaceutical approaches

Promising research is on the horizon to create treatments that aid in decreasing the severity or preventing, ID in DS patients.

Souchet et al. identified that green tea extract containing Epigallocathechin-3-Gallate (EGCG) is a natural inhibitor of Dyrk1a in adult mice overexpressing Dyrk1a or in Ts65Dn mice. It improved neurogenesis by enhancing the expression of B3-Tubulin and MAP2 [148, 149].

Other studies tried to test EGCC’s effect on humans with DS. A cohort study done by De la Torre et al. showed the effect of EGCC on 84 adults with DS. An experimental group was given an oral EGCC treatment daily (9 mg per kilogram of body weight), and the control group was given a placebo, for 1 year. This study was then integrated with a behavioral program of cognitive training for both groups. The experimental group showed a statistical advantage over the control group in two cognitive tasks and a handful of adaptive tasks (*p* < 0.05). Partial persistence of results was maintained 16 months later.

A study at UCLA [150] suggests that EGCG can also disaggregate tau proteins, multilayered filaments that form tangles that would kill neurons in the brain, ameliorating symptoms of Alzheimer’s and other amyloid diseases. They noted that EGCG is a poor therapeutic candidate due to its polyphenolic molecular structure restricting brain penetration. To resolve that issue, they treated the EGCG-binding position to tau proteins as a pharmacophore. They then computationally screened multiple drug-like compounds that could potentially be compatible with the pharmacophore. They identified various tau-disaggregating molecules with physiochemical drug-like properties superior to EGCG ones. A similar study could be done to identify the pharmacophore of EGCG that is specific in interacting with Dyrk1a to create potential drug-like compounds that better penetrate the brain of a DS patient to enhance their cognition.

Other potential perinatal therapies are Sonic Hedgehog (Shh) agonists and fluoxetine. Shh overexpression from the perinatal period protects the integrity of a DS brain and can enhance learning and memory in non-DS mice. In fact, it was shown that Ts65Dn mice that received a single injection of a Shh signaling agonist had a normalized cerebellar morphology and improved learning and memory [151]. Fluoxetine blocks serotonin reuptake in the presynaptic terminal by blocking the serotonin reuptake transporter present in the presynaptic terminal [152]. Guidi et al. also reported recovery of proliferation potency and cellularity of Ts65Dn mice treated with fluoxetine perinatally. In fact, Zhang et al. [152] conducted an experiment that showed that fluoxetine can enhance cognition in patients with Vascular Cognitive Impairment No Dementia (VCIND).

## Discussion

DS is the most common chromosomal abnormality, with an estimated incidence of 1 in 1000 to 1 in 1,1000 live births worldwide (UN, n.d.). Hence, enhancing our understanding of DS is necessary not only to improve therapeutic intervention methods but to do so early on. While over-inhibition appears to be an exciting hypothesis supporting the cognitive impairment seen in Ds patients, it is crucial to consider a more holistic approach that would allow us to recognize not only similarities between DS patients but also acknowledge the potential uniqueness of each DS patient.

Through understanding the morphology of pyramidal neurons, scientists were able to establish an association between abnormalities in dendritic spines and axons seen in DS patients with cognitive impairments. Scientists were also able to understand how the extra HSA21 copy can contribute to defective morphological presentation in these neurons through abnormal protein production. Studies have also pointed to potential abnormalities in LTP within the brains of DS patients and correlated such findings to GABAergic overexpression. The over-inhibition theory was also attributed to a potential elevation of GABA synapses rather than GABA concentrations. Research also pointed to elevated GABAergic interneurons in the hippocampus and cortex of Ts65Dn mice due to abnormality in the gene dosing of olig-1 and olig-2. Such findings indicate the need for more research to better understand the concept of over-inhibition.

Studies also pointed to reduced glutamatergic activity in DS patients, offsetting the balance between Glutamatergic and GABAergic activity. Such findings were attributed to a deficiency in the SNX27 gene. This implies that what may seem like a GABAergic overexpression can be due to an imbalance between GABAergic and glutamatergic activity.

Studies also determined an overexpression of several genes, such as DyrK1A, OLIG1/2, and MiRNA-155, whereas EURL, ERG, and SNX27 genes were reported to be underproduced. Through such findings, we can understand how complex it is to understand the exact cause of cognitive impairments in DS patients. However, such research has provided us with a gateway for better medical interventions as multiple approaches can be considered. Numerous experimental approaches can be considered to improve the neurological complications in DS brains and try to maintain optimal cognitive functioning. Such therapeutic approaches can involve chromosomal corrections via stem cell modification and pharmaceutical approaches to alter gene dosing.

## Conclusion

Down syndrome is a complex genetic disorder due to the complex pathophysiology accompanying the extra copy of chromosome 21. It is important to remember that DS does not present consistently but presents itself with a spectrum of challenges and difficulties. Therefore, by better understanding the pathophysiology of DS and by understanding the complexity of its presentation, huge medical advancements can be made for early intervention and support. Early intervention programs are necessary to maximize the positive outcomes in DS patients' physical, social, and cognitive development. Understanding how the extra copy of chromosome 21 leads to alterations in gene dosage, and the interactions of genes can help us better understand the varying manifestations and severity of DS features. The extra copy of chromosome 21 has also contributed to abnormalities in brain structure and neuronal functioning, contributing to varying levels of intellectual disabilities. Hence, through early intervention, such processes can be potentially halted to enhance the quality of life among DS patients. We can also better regulate and control the co-occurring conditions and ensure adequate medical care and support. DS is a pervasive disorder, and despite its debilitating effects on the patient, family unit, and society, its pathophysiology is indistinct. The key to understanding DS is by looking at it from a more holistic view, which this paper intends to offer.

## Data Availability

The authors confirm that the data presented in this study are available within the reference list of this article.
